# Shaping the Future of Nasal Surgery: A Systematic Review of Preservation Rhinoplasty

**DOI:** 10.1007/s00266-026-05853-9

**Published:** 2026-05-19

**Authors:** Dilan Gucuk, Leonard Knoedler, Tobias Niederegger, Jakob Fenske, Gabriel Hundeshagen, Max Heiland, Curtis L. Cetrulo, Olivier Mathieu, Thomas Schaschinger, Alexandre G. Lellouch

**Affiliations:** 1 Lausanne University Hospital (CHUV), University of Lausanne (UNIL), Lausanne, Switzerland; 2 Department of Oral and Maxillofacial Surgery, Charité–Universitätsmedizin Berlin, Corporate member of Freie Universität Berlin and Humboldt-Universität zu Berlin, Mittelallee 2, 13353 Berlin, Germany; 3 Medical Faculty Heidelberg, University of Heidelberg, Heidelberg, Germany; 4 Division of Plastic and Reconstructive Surgery, Cedars-Sinai Medical Center, Los Angeles, CA USA; 5 Vascularized Composite Allotransplantation Laboratory, Massachusetts General Hospital, Harvard Medical School, Boston, MA USA; 6 Innovative Therapies in Haemostasis, INSERM UMR-S 1140, University of Paris, 75006 Paris, France; 7 Department of Hand, Plastic and Reconstructive Surgery, Department of Plastic and Hand Surgery, Burn Center, BG Trauma Hospital Ludwigshafen, University of Heidelberg, Ludwigshafen, Germany

**Keywords:** Rhinoplasty, Preservation rhinoplasty, Dorsal rhinoplasty, Nasal surgery, Facial plastic surgery

## Abstract

**Background:**

Preservation rhinoplasty has transformed nasal surgery by prioritizing structural conservation while achieving aesthetic refinement and functional optimization. Unlike traditional rhinoplasty, which relies on extensive resection and reconstruction, preservation techniques aim to maintain key anatomical components, reducing complications and improving long-term stability. Recent advancements, including open dorsal preservation approaches and hybrid rhinoplasty techniques (bridging preservation and structural concepts), have expanded its indications, making it applicable to a broader range of nasal deformities and functional impairments. However, the efficacy, limitations, and functional outcomes of these techniques remain to be synthesized.

**Methods:**

A systematic review was conducted following PRISMA 2020 guidelines, searching PubMed/MEDLINE, EMBASE, and Web of Science databases up to March 1^st^, 2025. Studies evaluating surgical techniques, functional and aesthetic outcomes, complications, and patient satisfaction in preservation rhinoplasty were included. Given the heterogeneity of study designs and outcome measures, a narrative synthesis was performed.

**Results:**

Thirteen studies comprising 855 patients were included, with publication years ranging from 2020 to 2024 and a mean Newcastle–Ottawa Scale score of 5.8 (SD 0.4), indicating moderate methodological quality. Dorsal preservation techniques—including push-down, let-down, and subdorsal strip methods—consistently yielded favorable results. Patient satisfaction was high, with Rhinoplasty Outcome Evaluation (ROE) scores exceeding 85 in up to 90.3% of patients. Functional improvement was reported across techniques, with increased nasal patency scores and reduced Nasal Obstruction Symptom Evaluation (NOSE) scores. Hybrid approaches such as mix-down and Low Strip Unifying Hybrid Rhinoseptoplasty (LUHRS) were especially beneficial in complex nasal anatomies, with RHINO scores rising from 37.6 to 88.2. Reported revision rates across all approaches ranged from 0 to 7.9%, typically addressing minor contour irregularities without major complications.

**Conclusion:**

Preservation rhinoplasty has emerged as a potentially promising alternative to traditional structural techniques and may offer favorable dorsal contour preservation, low reported complication rates, and high levels of patient satisfaction in selected patients. Careful patient selection appears to remain essential, with ideal candidates often presenting with straight nasal profiles, minimal dorsal deviation, and relatively intact nasal anatomy. While early outcomes are encouraging, the predominance of observational data, limited use of standardized objective functional assessments, and relatively short follow-up periods restrict definitive conclusions regarding long-term durability and comparative effectiveness. Future research should aim to further refine patient selection criteria, clarify the role of hybrid techniques in more complex cases, and explore the integration of technologies such as 3D imaging and artificial intelligence to potentially enhance surgical planning and outcome assessment in modern nasal surgery.

**Level of Evidence II:**

This journal requires that authors assign a level of evidence to each article. For a full description of these Evidence-Based Medicine ratings, please refer to the Table of Contents or the online Instructions to Authors www.springer.com/00266.

**Graphical Abstract:**

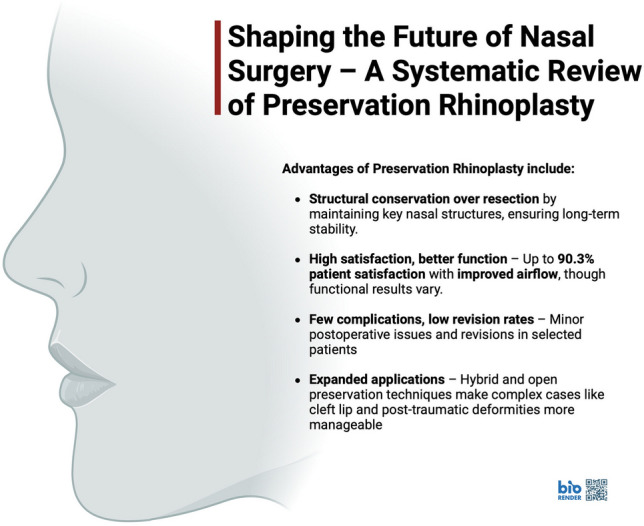

**Supplementary Information:**

The online version contains supplementary material available at 10.1007/s00266-026-05853-9.

## Introduction

Rhinoplasty is a complex and evolving field within facial plastic surgery, requiring a delicate balance between aesthetics, function, and structural longevity [1, 2]. While traditional structural rhinoplasty techniques are considered the standard for correcting nasal deformities and functional impairments, they often involve extensive tissue resection, which can lead to complications such as dorsal irregularities, prolonged healing, and long-term instability. In response to these challenges, preservation rhinoplasty has emerged as a technique that prioritizes the maintenance of key anatomical structures while achieving natural and lasting results. [3–7]

The preservation rhinoplasty technique originated in the early twentieth century, first described by Drs. Goodale, Lothrop, and Cottle [8, 9]. However, it has gained widespread recognition in recent years, largely due to the innovative contributions of Dr. Dogan [5, 6, 10–12]. Among its approaches, dorsal preservation rhinoplasty has attracted particular attention for its ability to conserve the nasal dorsum and maintain natural contour lines. Various modifications, including the subdorsal strip method, let-down and push-down techniques, and hybrid rhinoplasty techniques, have been introduced to enhance surgical precision and optimize patient outcomes [13–15]. These techniques offer promising advantages, such as reduced complications, improved long-term stability, compared to traditional hump reduction approaches [4, 5, 16].

The use of preservation rhinoplasty across different patient demographics and nasal anatomies is an area of ongoing research. Patient selection remains crucial, as certain anatomical variations may not be ideal candidates for this approach [11, 17]. Additionally, preservation techniques are increasingly being applied to male rhinoplasty, where maintaining strong, masculine nasal features while improving function is a priority [18]. Furthermore, novel approaches, including the mix-down and semi-let-push-down techniques, have expanded their applicability to complex cases such as cleft lip nasal deformities [19].

Despite the advancements in preservation rhinoplasty, further research is necessary to refine patient selection criteria, assess long-term outcomes, and expand indications for its use. This study aims to contribute to this growing body of knowledge by evaluating the latest developments in preservation rhinoplasty techniques, assessing their efficacy, and exploring their role in shaping the future of nasal surgery.

## Methods

This systematic review aligned with the Preferred Reporting Items for Systematic Reviews and Meta-Analyses (PRISMA) 2020 guidelines. Given the heterogeneity in data, a meta-analysis was considered unsuitable, and a narrative synthesis was performed. To ensure clarity and avoid confusion of concepts, in this review, we use preservation rhinoplasty as the umbrella term; dorsal preservation refers specifically to techniques that maintain the dorsal roof (e.g., push-down/let-down/subdorsal strip concepts); and hybrid rhinoplasty denotes approaches that combine preservation principles with selective structural maneuvers when additional support or correction is required.

### Systematic Search

The comprehensive literature search was conducted to evaluate the advancements and clinical outcomes of preservation rhinoplasty. The search encompassed the PubMed, EMBASE, and Web of Science databases until March 1^st^, 2025. The search strategy included the following keywords: "preservation rhinoplasty," "dorsal preservation," "hybrid rhinoplasty," "let-down technique," "push-down technique," and "structural rhinoplasty." Boolean operators (AND, OR) were applied to refine the search, ensuring the inclusion of relevant studies. Additionally, relevant studies were cross-referenced to enhance the depth and completeness of the analysis.

Inclusion criteria were I) clinical studies evaluating preservation rhinoplasty techniques, II) comparative studies between structural and preservation approaches, III) systematic reviews and meta-analyses on nasal dorsum preservation, IV) studies discussing long-term functional and aesthetic outcomes. Exclusion criteria included I) case reports with fewer than five patients, II) non-English articles, III) studies lacking sufficient postoperative follow-up.

Two independent reviewers (D.G., J.F.) performed abstract and full text screening. If discrepancies occurred, a third senior investigator (A.G.L.) was consulted, resolving discrepancies through discussion.

**Fig. 1:**
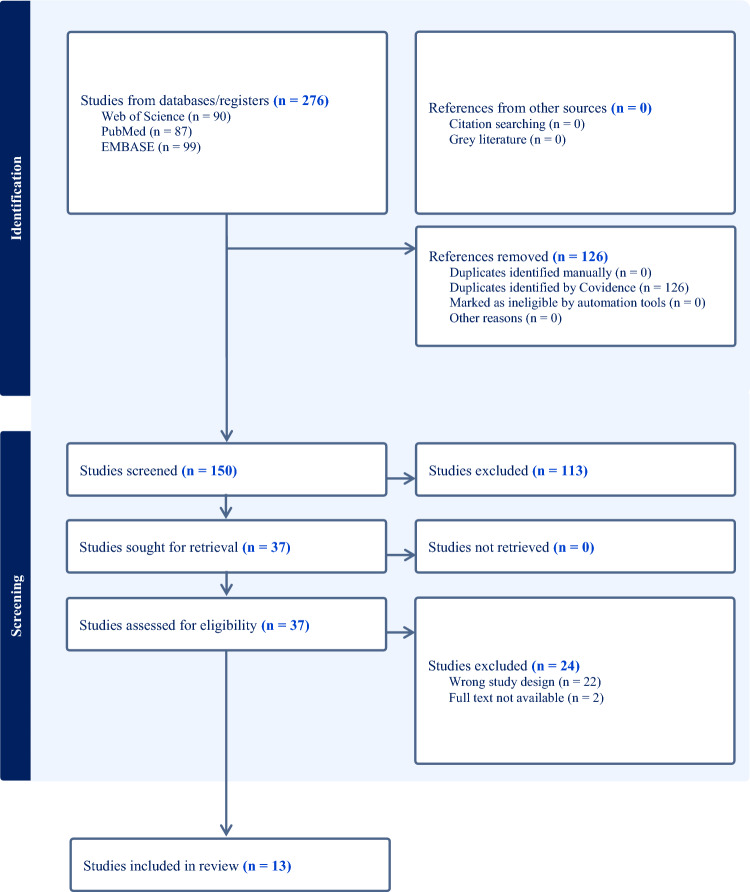
PRISMA 2020 flow chart highlighting the study workflow

The complete search strategy for each database is provided in Supplementary Digital Content 1, while Figure 1 presents the PRISMA 2020 flowchart, outlining the study selection process.

### Quality Assessments

The quality of the studies was evaluated using the Newcastle–Ottawa Scale (NOS) and the Level of Evidence (LOE) framework. The NOS assessed three main domains, assigning up to nine stars per study: I) selection of study cohorts (maximum of four stars), II) comparability of study groups (maximum of two stars), and III) assessment of outcome or exposure measurements (maximum of three stars). A higher NOS score indicated better study quality and a lower risk of bias.

**Fig. 2:**
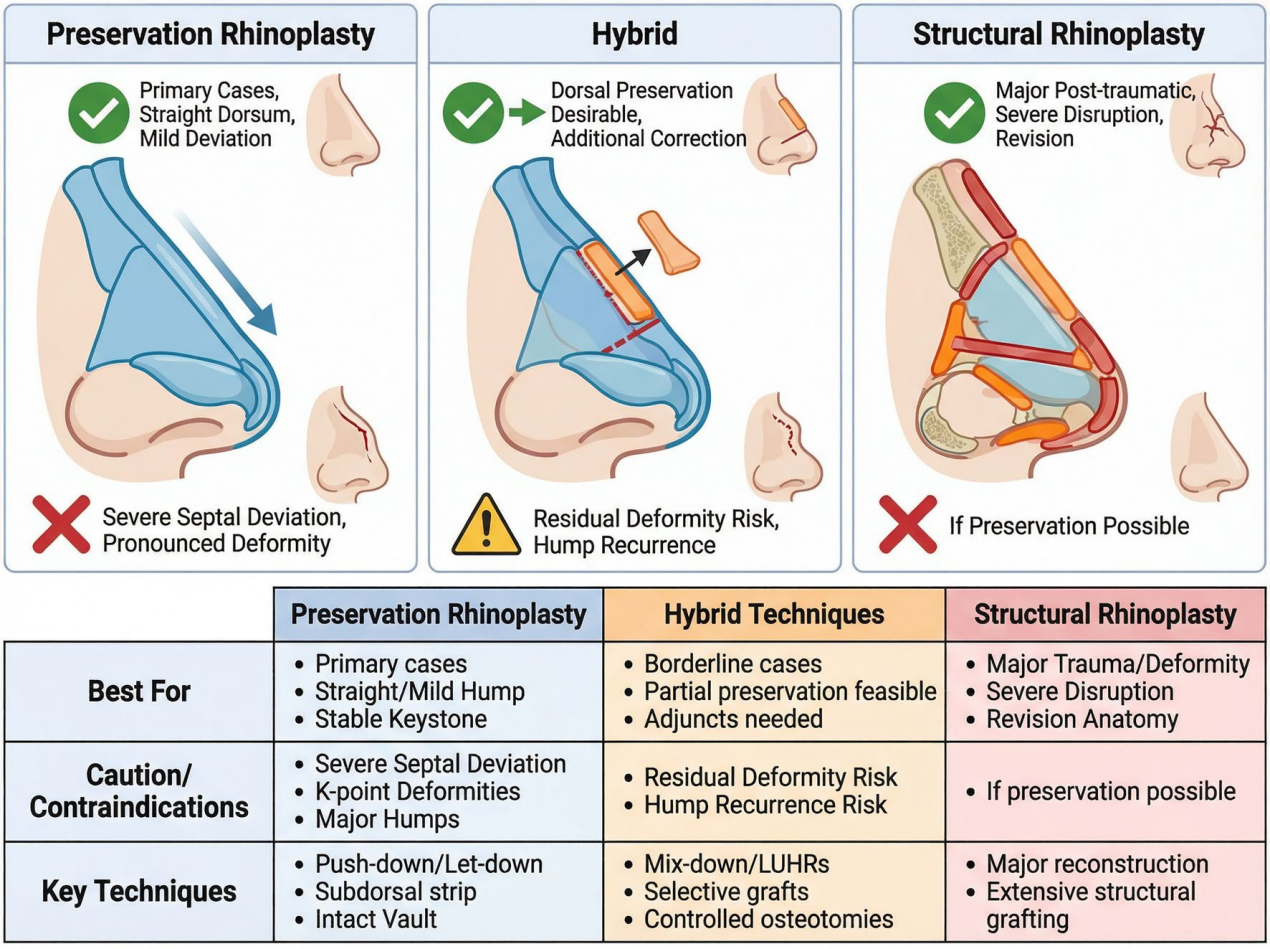
Comparative overview of preservation, hybrid, and structural rhinoplasty approaches

The LOE classification was based on the methodological rigor and potential for bias in different study designs, establishing a hierarchical ranking. Studies with strong methodology, such as systematic reviews and meta-analyses derived from well-executed randomized controlled trials (RCTs), were classified as LOE I, representing the highest level of evidence (Fig. 2).

### Data Extraction

Following the blinded, double-review process, we extracted the following parameters: Digital Object Identifier, first author, study title, year of publication, study design, sample size, length of follow-up, surgical technique, aesthetic and functional outcomes, postoperative complications, and patient- and surgeon-reported satisfaction scores.

A quantitative aggregation of functional and aesthetic outcomes (e.g., ROE/NOSE) was considered; however, this was not feasible due to substantial heterogeneity in reporting instruments (e.g., ROE 0–100, ROE 0–30, NOSE, RHINO, internal nasal valve angle, absence of paired means or variance, and marked clinical heterogeneity across studies.

## Results

The systematic database search retrieved a total of 276 studies, of which *n *= 13 (4.7%) met the predefined inclusion and exclusion criteria. The mean (SD) NOS was 5.8 (0.4), suggesting an overall moderate methodological quality. Year of publication ranged from 2020 to 2024. In summary, 855 patients were included. Follow-up durations ranged from six to 28 months. Further details on patient demographics and quality assessments are provided in Table 1, Supplementary Digital Content 2 and 3. Study-level ROE, NOSE, and RHINO outcomes are reported individually when available.

**Table 1:** Patient demographics

Author	DOI	Title	Year of publication	Study design	Sample size	Length of follow-up	Surgical technique
Öztürk et al.	10.1007/s00266-020-01660-y	Push-Down Technique Without Osteotomy: A New Approach	2020	Cohort study	62	12–18 months (median: 14.2 months)	Modified push-down technique without osteotomy, using micromotor rasping instead
Kosins et al.	10.1093/asj/sjz107	Decision Making in Preservation Rhinoplasty: A 100 Case Series With One-Year Follow-Up	2020	Cohort study	153	At least 12 months (mean: 13 months)	Partial and complete preservation rhinoplasty with preservation of the soft tissue envelope and ligaments, as well as preservation of alar cartilage and dorsal preservation
Ferraz et al.	10.1055/s-0041-1725154	Indications for Preservation Rhinoplasty: Avoiding Complications	2021	Original article	N/A	N/A	Preservation rhinoplasty techniques, including SPAR (Septum Pyramidal Adjustment and Repositioning), push-down, and let-down techniques
Öztürk	10.1093/asj/sjaa436	Semi–Let-Down and Semi–Push-Down Preservation Techniques: Maintaining the Intactness of the Distal Region	2021	Cohort study	64	16–25 months (median: 19.2 months)	Closed rhinoplasty with push-down and let-down techniques
Tuncel et al.	10.1093/asj/sjac069	Dorsal Preservation Surgery: A Novel Modification for Dorsal Shaping and Hump Reduction	2022	Cohort study	25 (22 primary, 3 secondary rhinoplasty cases)	6–19 months (mean: 10.3 months)	Dorsal Roof Flap (DRF) technique with two variations: cartilaginous DRF and osseocartilaginous DRF
Öztürk et al.	10.1097/SCS.0000000000008553	Hybrid Preservation Rhinoplasty: Combining Mix-Down and Semi Let-Push Down Techniques	2022	Cohort study	36	13–28 months (median: 19.8 months)	Hybrid preservation rhinoplasty, combining mix-down, semi-let-down, and semi-push-down techniques
Kosins	10.1093/asj/sjac074	Preservation Rhinoplasty: Open or Closed?	2022	Cohort study	162	At least 1 year	Open and closed (with preservation of dorsal soft tissue envelope) rhinoplasty. Dorsal preservation with surface or impaction technique. Structural rhinoplasty with piezoelectric osteotomies and mid-vault reconstruction
Stergiou et al.	10.1016/j.bjps.2021.05.073	A multivariate analysis after preservation rhinoplasty (PR) - a prospective study	2022	Prospective study	30	1–18 months (mean: 8.4 months)	Preservation rhinoplasty techniques, including push-down, and let-down techniques
Urs et al.	10.37897/RMJ.2022.2.7	Risks and complications in rhinoplasty. A comparative study in structural vs preservation rhinoplasty	2022	Cohort study	100	N/A	50 patients with structural/traditional rhinoplasty, 50 patients with preservation rhinoplasty (PR) (25 with push-down, 25 with let-down technique). All surgeries included rhinoseptoplasty
Tapan et al.	10.1097/SAP.0000000000003681	Let-Down Rhinoplasty in Patients With Cleft Lip Nose	2023	Cohort study	10	12 months	Let-down rhinoplasty open or closed with costal cartilage an full-thickness skin grafts
Anco et al.	10.1097/GOX.0000000000004972	Preservation Rhinoplasty: A New Approach to Mestizo Noses	2023	Cohort study	14	N/A	Preservation rhinoplasty techniques, including push-down, and let-down techniques
Scafati et al.	10.1097/PRS.0000000000011307	Low Strip Unifying Hybrid Rhinoseptoplasty: A Novel Classification in Dorsal Preservation Surgery	2024	Cohort study	190	12 months	Low Strip Unifying Hybrid Rhinoseptoplasty (LUHRS), a hybrid preservation–structural approach involving dorsal preservation with modifications like push-down, let-down, LUHRS I-V techniques
Jin et al.	10.1097/PRS.0000000000010472	Dorsal Preservation Rhinoplasty in Asian Hump Noses	2024	Retrospective case series	9	At least 6 months (mean: 8.3 months)	Hump reduction using open approach with push-down or let-down techniques. Tip modification with or without radix grafting

*DOI* Digital object identifier, *N/A* Not applicable

### Patient Selection, Outcome Assessment, and Surgical Results

Patient selection was a key factor in determining the surgical success of preservation rhinoplasty. Studies mainly focused on individuals with straight nasal profiles, intact dorsal anatomy, and limited septal deviation—factors that are critical for maintaining structural stability when using dorsal preservation techniques. Favorable soft tissue characteristics and patient expectations regarding functional and aesthetic outcomes also played a role in guiding the choice of surgical approach.

Aesthetic and functional outcomes were assessed using patient-reported instruments (e.g., ROE, NOSE, RHINO) and, in some studies, objective parameters such as nasal patency visual analog scale (VAS) or internal nasal valve angle. Öztürk et al. investigated a modified push-down technique without osteotomy in a cohort of 62 patients (median follow-up: 14.2 months), reporting significant improvements in nasal dorsum contour and high satisfaction, with 90.3% of patients scoring above 85 on the Rhinoplasty Outcome Evaluation (ROE) scale. The ROE is a six-item, patient-completed questionnaire that assesses satisfaction with nasal function and appearance, with scores converted to a 0–100 scale—higher values indicating greater satisfaction. In their study, the median postoperative ROE score was 90.5, reflecting strong subjective outcomes. Objective functional improvements were also noted, with nasal patency scores increasing from 6 to 8 out of 10. The nasal patency score is a simple VAS ranging from 0 (complete obstruction) to 10 (perfectly clear nasal breathing), used to subjectively quantify nasal airflow as perceived by the patient. No complications or revision surgeries were reported.

Similarly, Jin et al. assessed functional outcomes using the Nasal Obstruction Symptom Evaluation (NOSE) scale, a five-item questionnaire quantifying nasal obstruction on a 0–100 scale, where higher scores represent more severe symptoms. All nine patients in their cohort showed improvement, with a mean NOSE score reduction from 45.6 to 18.4, indicating substantial enhancement in nasal airflow following dorsal preservation rhinoplasty.

Furthermore, Kosins et al. compared partial and complete preservation rhinoplasty, emphasizing the importance of maintaining the soft tissue envelope, alar cartilage, and nasal ligaments to ensure structural integrity. While functional outcomes were not comprehensively assessed, their study documented three surgical revisions and one case of submucosal septal infection.

Ferraz et al. evaluated the effectiveness of Septum Pyramidal Adjustment and Repositioning (SPAR), push-down, and let-down techniques, demonstrating that these preservation approaches maintain a natural nasal dorsum shape while protecting the keystone area and nasal valve. However, the study highlighted potential complications, including radix step, residual dorsal humps, widening of the middle third, and saddle deformity, particularly in cases where surgical indications were not well defined.

Tuncel et al. assessed the Dorsal Roof Flap (DRF) technique, a modification aimed at optimizing dorsal shaping and hump reduction. Their findings indicated a more natural nasal dorsum, well-defined dorsal aesthetic lines, and a significant improvement in the nasolabial angle. Additionally, no complications, residual humps, or revision surgeries were reported, suggesting strong long-term stability.

At last, Stergiou et al. reported that preservation rhinoplasty resulted in an increased internal nasal valve angle and widened nasal cavity, indicating a potential functional benefit in nasal airflow improvement. However, minor complications such as hematomas, wound dehiscences, and nasal tip infections were noted, with two patients requiring revision surgery.

Across all studies, patient satisfaction was consistently high, with most reporting improvements in nasal aesthetics and functional breathing. Studies by Öztürk et al., Scafati et al., and Tapan et al. demonstrated significant postoperative increases in ROE and Rhinoplasty Health Inventory and Nasal Outcomes (RHINO) scores, reflecting strong subjective approval of surgical outcomes. The RHINO score is a validated, patient-reported outcome measure that assesses both functional and aesthetic satisfaction after rhinoplasty, scaled from 0 to 100, with higher scores indicating better overall nasal health and satisfaction.

Scafati et al. noted enhancements in nasal airflow, while Öztürk et al. reported high satisfaction with hybrid approaches. The low revision rates across studies, ranging from 0 to 7.9%, further supported the long-term stability and reliability of preservation rhinoplasty compared to traditional structural techniques. Scafati et al. reported a 7.9% revision rate (15 out of 190 patients), mostly for minor residual humps and nostril corrections, while Tuncel et al. observed no need for revision surgeries in their cohorts. Stergiou et al. reported two revision procedures for minor complications in a series of 30 patients. Comprehensive information is provided in Table 2.

**Table 2:** Overview on functional and aesthetic outcomes, complications as well as surgeon and patient satisfaction

Author	DOI	Title	Functional outcome	Aesthetic outcome	Complications	Surgeon satisfaction	Patient satisfaction
Öztürk et al.	10.1007/s00266-020-01660-y	Push-Down Technique Without Osteotomy: A New Approach	Improved nasal airflow, with an increase in patency score from 6 to 8 (out of 10)	Significant improvement in nasal dorsum contour, high patient satisfaction (90.32% scored >85 on the ROE scale)	No complications or need for revision surgery reported	N/A	High satisfaction, with a postoperative median Rhinoplasty Outcome Evaluation (ROE) score of 90.5
Kosins et al.	10.1093/asj/sjz107	Decision Making in Preservation Rhinoplasty: A 100 Case Series With One-Year Follow-Up	N/A	N/A	3 surgical revisions, 1 submucosal septal infection	N/A	N/A
Ferraz et al.	10.1055/s-0041-1725154	Indications for Preservation Rhinoplasty: Avoiding Complications	Helps preserve the keystone area and nasal valve, reducing risks of nasal obstruction	Maintains natural nasal dorsum shape; can result in a lower radix, increased interalar distance, and a wider middle third	Potential issues include radix step, residual hump, widening of the middle third, saddle deformity, and functional problems if not properly indicated	N/A	N/A
Öztürk	10.1093/asj/sjaa436	Semi–Let-Down and Semi–Push-Down Preservation Techniques: Maintaining the Intactness of the Distal Region	Satisfactory by subjective measures	Satisfactory by subjective measures	N/A	N/A	Excellent satisfaction according to Rhinoplasty Outcome Evaluation questionnaire
Tuncel et al.	10.1093/asj/sjac069	Dorsal Preservation Surgery: A Novel Modification for Dorsal Shaping and Hump Reduction	Not explicitly detailed	Achieved a more natural nasal dorsum with well-defined dorsal aesthetic lines; statistically significant improvement in the nasolabial angle	No reported complications, residual humps, or revision surgeries	N/A	Positive results with no postoperative hump deformities and good long-term dorsal stability
Öztürk et al.	10.1097/SCS.0000000000008553	Hybrid Preservation Rhinoplasty: Combining Mix-Down and Semi Let-Push Down Techniques	Observed improvement of breathing by subjective measures	Satisfactory by subjective measures	2 minor hump recurrences without need for revision	Satisfactory by subjective measures	Excellent satisfaction according to Rhinoplasty Outcome Evaluation questionnaire
Kosins	10.1093/asj/sjac074	Preservation Rhinoplasty: Open or Closed?	Not comprehensively analyzed	Not comprehensively analyzed	Not comprehensively analyzed	N/A	Not comprehensively analyzed
Stergiou et al.	10.1016/j.bjps.2021.05.073	A multivariate analysis after preservation rhinoplasty (PR) - a prospective study	Widening of nasal cavity with increased internal nasal valve angle	N/A	Seven minor complications (three hematomas, three wound dehiscences, one nasal tip infection). Two revision surgeries.	N/A	High satisfaction according to Rhinoplasty Outcome Evaluation questionnaire
Urs et al.	10.37897/RMJ.2022.2.7	Risks and complications in rhinoplasty. A comparative study in structural vs preservation rhinoplasty	N/A	N/A	3 septal hematomas (1 in PR), 10 epistaxis (3 in PR), 10 epiphora (3 in PR), 30 dental trauma (10 in PR), 5 nasal septal perforations (1 in PR), 10 pollybeaks (2 in PR), 9 saddle noses (0 in PR), 10 middle vault asymmetries (0 in PR), 15 nasal tip overprojection (o in PR), 12 nasal tip underprojection (0 in PR), 10 tip asymmetries (2 in PR), 7 alar retractions (0 in PR), 10 surgical revisions (2 in PR), 10 internal nasal valve dysfunctions (0 in PR), 8 patients with external nasal valve dysfunction (0 in PR), 3 collapse alar (0 in PR), 5 residual anterior septal deviations (0 in PR)	N/A	N/A
Tapan et al.	10.1097/SAP.0000000000003681	Let-Down Rhinoplasty in Patients With Cleft Lip Nose	Satisfactory by subjective measures	Satisfactory by subjective measures	N/A	N/A	Increased post- to preoperative satisfaction according to Rhinoplasty Outcome Evaluation questionnaire
Anco et al.	10.1097/GOX.0000000000004972	Preservation Rhinoplasty: A New Approach to Mestizo Noses	N/A	N/A	N/A	N/A	Increased post- to preoperative satisfaction according to Rhinoplasty Outcome Evaluation questionnaire
Scafati et al.	10.1097/PRS.0000000000011307	Low Strip Unifying Hybrid Rhinoseptoplasty: A Novel Classification in Dorsal Preservation Surgery	Significant improvement in nasal airflow, with RHINO score increasing from 37.6 preoperatively to 88.2 postoperatively	Improved nasal dorsum shape, smooth dorsal aesthetic lines, high patient satisfaction	7.89% revision rate (15/190 patients), primarily for minor residual humps and nostril corrections, no major complications or septal perforations	N/A	High satisfaction, significant postoperative RHINO score increase
Jin et al.	10.1097/PRS.0000000000010472	Dorsal Preservation Rhinoplasty in Asian Hump Noses	Improved Nasal Obstruction Symptom Evaluation scale in all patients	Decrease in nasofacial angle and rhinion angle, indicating hump reduction. No changes in nasofrontal and nasolabial angle.	No major complications	N/A	Satisfactory by subjective measures concerning aesthetics and function

*DOI* Digital object identifier, *N/A* Not applicable, *PR* preservation rhinoplasty, *RHINO* Rhinoplasty Health Inventory and Nasal Outcomes

### Preservation of Dorsal Aesthetic Lines and Nasal Framework Integrity

Öztürk et al. reported favorable outcomes using semi-let-down and semi-push-down techniques via a closed approach, with stable nasal contours and high ROE-based patient satisfaction. Scafati et al. evaluated the Low Strip Unifying Hybrid Rhinoseptoplasty (LUHRS)—a hybrid technique—and observed significant improvements in nasal airflow, with RHINO scores increasing from 37.6 to 88.2. A 7.9% revision rate was noted in their study, primarily for minor contour adjustments, with no major complications reported.

Kosins et al. compared open and closed methods, while Tapan et al. used an open let-down approach in cleft lip nose patients, achieving satisfactory outcomes. Similarly, Jin et al. employed an open preservation approach in Asian hump noses, reporting improved NOSE scores and effective hump reduction without major complications.

Hybrid rhinoplasty techniques (preservation–structural) have also proven beneficial in anatomically challenging cases. Öztürk et al. combined mix-down, semi-let-down, and semi-push-down techniques, achieving subjective improvements in breathing with only two minor, non-revised hump recurrences. Urs et al. compared structural and preservation approaches, finding fewer complications—such as septal perforation, pollybeak, and tip projection issues—in the preservation group.

Overall, both open and closed preservation rhinoplasty approaches yield favorable outcomes, with a growing trend toward open techniques in complex cases. Further details are provided in Table 2*.*

## Discussion

This study explored the evolution of preservation rhinoplasty techniques, analyzing their advantages, limitations, and clinical applications. By reviewing contemporary literature and assessing the outcomes associated with preservation approaches, we aimed to evaluate their impact on surgical results, patient satisfaction, and long-term nasal structure stability.

The findings underscore the growing preference for preservation rhinoplasty, reflecting its advantages in maintaining anatomical integrity while reducing complications associated with traditional structural techniques. Furthermore, this study highlights key innovations that have shaped the field, offering insight into the future trajectory of rhinoplasty advancements.

### Long-Term Functional Stability and Airway Patency

One of the most significant findings in this review is the potential functional benefits of preservation rhinoplasty, particularly in maintaining nasal airflow and internal valve integrity. Several studies reported improvements in nasal patency scores and widening of the nasal cavity, suggesting that preservation techniques could provide better long-term functional stability compared to traditional structural approaches. By maintaining the keystone area and limiting disruption to the dorsal framework, preservation rhinoplasty theoretically reduces the risk of long-term airway compromise. However, while short-term outcomes are promising, the long-term durability of these functional improvements remains an open question [20].

Late-onset complications such as internal valve collapse, persistent nasal obstruction, and dorsal irregularities were not extensively assessed in the included studies, highlighting a critical gap in current literature. Future research should aim to incorporate standardized, objective assessments of nasal airflow over extended follow-up periods to evaluate whether preservation techniques offer superior long-term functional benefits [21, 22]. Methods such as computational fluid dynamics analysis, acoustic rhinometry, and objective airflow measurements could provide valuable insights into the efficacy of these techniques over time. Additionally, identifying patient-specific factors, such as pre-existing nasal valve abnormalities or septal deviations, that may predispose individuals to functional decline postoperatively could help refine patient selection criteria for preservation rhinoplasty [23].

### Patient Satisfaction Following Preservation Rhinoplasty

Patient satisfaction is a key metric in evaluating the success of any rhinoplasty technique [24], and the included studies consistently reported high satisfaction rates with preservation rhinoplasty. The significant postoperative improvements in ROE and RHINO scores reflect both aesthetic and functional benefits perceived by patients. The high satisfaction rates may be attributed to the ability of preservation techniques to maintain natural dorsal aesthetic lines while minimizing complications such as dorsal irregularities and tip deformities.

However, patient satisfaction extends beyond the immediate surgical outcome and is influenced by various psychological and quality-of-life factors. Preoperative expectations, perception of nasal function, and overall confidence following surgery play a role in determining how patients evaluate their results. While preservation rhinoplasty appears to provide a more predictable and natural aesthetic outcome, long-term patient-reported outcomes remain underexplored. Further research should investigate the psychological impact of preservation rhinoplasty, assessing parameters such as postoperative self-esteem, body image satisfaction, and long-term quality of life [25]. Additionally, employing artificial intelligence (AI)-based tools to objectively quantify pre- and postoperative results, could complement the subjective perception and aid in patient guidance [2, 26, 27].

### Revision Rate and Need for Secondary Procedures

One of the key advantages of preservation rhinoplasty reported across the included studies is its low revision rate. Major complications requiring surgical revision were rare, and minor revisions—when performed—typically addressed small contour irregularities or nostril asymmetries. This suggested that preservation techniques may offer enhanced predictability and long-term stability in surgical outcomes.

By comparison, traditional structural rhinoplasty has been associated with significantly higher revision rates, often cited in the literature as ranging from 5 to 15%, and in some reports, up to 20–25% depending on the patient population, technique, and surgeon experience [28, 29]. For example, Neaman et al. reported a 9.8% revision rate in a series of 369 primary cosmetic rhinoplasties, with dissatisfaction and complications closely linked to unaddressed anatomical issues [30]. Similarly, Bouaoud et al. found a revision rate of 8.6% in a study of 732 closed rhinoplasties, identifying pre-existing functional disorders and wide nasal bones as predictors for revision need [31]. Although minor imperfections such as residual dorsal humps, radix step deformities, or middle vault widening were occasionally reported in preservation cases, they were typically well-tolerated or corrected with minimal intervention. One of the most common and specific complications of dorsal preservation rhinoplasty is the recurrence of the dorsal hump, with incidence rates ranging from 1% to 15% and often requiring secondary intervention [10, 32–35]. This complication can be attributed to two distinct mechanisms: residual humps, due to incomplete resection or septal modification, and recurrence, resulting from persistent tensile forces or “position memory” of the osseocartilaginous vault [36]. To mitigate this risk, Cason et al. have proposed five critical strategies: (1) meticulous patient selection, (2) comprehensive release of all eight anatomic blocking points [37], (3) mechanical fixation of the cartilaginous vault through suture techniques tailored to the type of septal resection [10, 34, 38, 39], (4) a graduated approach to procedural selection based on dorsal morphology, and (5) ancillary maneuvers such as pyriform ligament release to reduce deforming forces at the keystone area [40]. Implementation of these principles has been shown to reduce the risk of hump recurrence and improve overall stability in preservation rhinoplasty cases. In addition to skeletal structures, soft tissue elements—particularly the Pitanguy ligament—may also contribute to dorsal instability and recurrence if not properly addressed [41]. Traditionally preserved in closed preservation rhinoplasty, the Pitanguy midline ligament can exert downward tension on the nasal tip and supratip region [41]. Recent cadaveric studies have demonstrated that in cases of tip rotation or reduction, the native position of this ligament may become incongruous with the reshaped nasal framework [41]. If left untreated, its tethering effect may contribute not only to supratip fullness or depression but also to dorsal contour irregularities. Selective release and cephalad repositioning of the Pitanguy ligament have been proposed as an effective adjunct to improve soft tissue redraping, enhance tip definition, and potentially reduce the likelihood of late dorsal hump recurrence. These findings underscore the importance of considering both structural and soft tissue dynamics when planning preservation-based approaches. When compared to conventional approaches, these findings support the notion that preservation rhinoplasty may reduce the need for secondary procedures, offering both aesthetic and functional reliability with fewer long-term complications.

The need for revision procedures in preservation rhinoplasty, though relatively low, raises important questions regarding patient selection and surgical technique refinement. Hybrid approaches that integrate preservation principles with structural modifications appear to provide additional flexibility in complex cases but may also introduce a slightly higher likelihood of requiring minor adjustments. Future research should aim to identify specific risk factors associated with revision surgery in preservation rhinoplasty, including anatomical variations, surgeon experience, and technique selection [42].

Additionally, establishing standardized protocols for managing minor postoperative imperfections is essential to optimizing patient outcomes. Developing minimally invasive secondary procedures tailored to preservation rhinoplasty patients could help address minor irregularities while maintaining the benefits of the initial surgery. For example, injectable fillers could be explored as **a** viable option for postoperative refinements [22].

### Potential Indications, Relative Contraindications, and When to Consider Hybrid/Structural Techniques

Preservation rhinoplasty is most predictable when native dorsal and septoligamentous anatomy can be maintained without forcing major realignment. Accordingly, ideal candidates are typically primary cases with a straight or mildly convex dorsal profile, minimal to moderate dorsal deviation, a stable keystone area, intact upper lateral cartilages, and limited septal pathology, where controlled lowering of the osseocartilaginous vault (e.g., push-down/let-down/subdorsal strip concepts) can correct the aesthetic concern while preserving dorsal aesthetic lines and valve function. In contrast, several relative contraindications should prompt heightened caution or an alternative strategy: severe septal deviation (particularly involving the dorsal septum/keystone), pronounced K-point deformities, marked asymmetric bony pyramid, significant post-traumatic distortion with loss of dorsal “en bloc” mobility, substantial pre-existing valve compromise requiring robust reconstruction, and scenarios in which dorsal lowering alone cannot address the underlying framework imbalance (e.g., complex twisting, major humps with rigid or irregular bony–cartilaginous interfaces). In these settings, outcomes may be less predictable and the risk of residual deformity, hump persistence/recurrence, or midvault irregularities may increase. Therefore, hybrid approaches, integrating preservation principles with targeted structural maneuvers (e.g., selective septoplasty, limited grafting/spreader support, controlled osteotomies, or techniques such as mix-down/LUHRS concepts), are often preferable when dorsal preservation is desirable, but additional stabilization or correction is required. Finally, fully structural rhinoplasty may be favored when there is a clear need for extensive rebuilding (e.g., major post-traumatic deformity, severe septal/keystone disruption, or significant revision anatomy), where predictable alignment and durable support outweigh the advantages of maximal preservation (Table 3) [43, 44].

**Table 3:** Indications, relative contraindications, and when to consider hybrid/structural techniques

Category	Key features/Clinical scenarios	Examples/Notes
Potential Indications for Preservation Rhinoplasty	Cases where native dorsal and septoligamentous anatomy can be maintained without major realignment	- Primary rhinoplasty (first-time surgery) - Straight or mildly convex dorsal profile - Minimal to moderate dorsal deviation - Stable keystone area - Intact upper lateral cartilages - Limited septal pathology - Goal: controlled dorsal lowering (push-down, let-down, subdorsal strip) to correct aesthetic concern while preserving dorsal lines and valve function
Relative Contraindications	Conditions that increase risk of unpredictable results or require structural correction	- Severe septal deviation (especially dorsal/keystone involvement) - Pronounced K-point deformities - Marked asymmetric bony pyramid - Significant post-traumatic distortion with loss of dorsal mobility - Pre-existing valve compromise needing strong reconstruction - Complex twisting or major humps with rigid/irregular interfaces - Scenarios where dorsal lowering alone won’t address imbalance
When to Consider Hybrid/Structural Approaches	When preservation alone cannot achieve stable alignment or adequate correction	- Need for additional support or reconstruction - Minor structural additions while maintaining preservation goals - Techniques: selective septoplasty, limited grafting or spreader support, controlled osteotomies, “mix-down” or LUHRS methods - Hybrid approaches suit mild–moderate complexity - Fully structural rhinoplasty preferred for major trauma, keystone/septal loss, or challenging revisions

### Conceptual Positioning of Hybrid Techniques

Hybrid rhinoplasty techniques may be more appropriately understood not as a subset of preservation rhinoplasty alone, but rather as a conceptual and technical bridge between pure preservation and fully structural approaches. While they retain core preservation principles, such as dorsum conservation and respect for native ligamentous and septal anatomy, they simultaneously incorporate selective structural maneuvers when additional correction, stabilization, or reconstruction is necessary. In this sense, hybrid approaches expand the surgical spectrum by allowing graded intervention tailored to anatomical complexity, rather than adhering strictly to either a preservation-only or reconstruction-dominant philosophy. Framing hybrid techniques in this way may help clarify their role in borderline or complex cases, where complete preservation may be insufficient, yet a fully structural rebuild may be unnecessarily aggressive.

### Balance Between Patient-Reported Outcomes and Objective Airflow Measurements

An important consideration when interpreting the reported functional benefits of preservation rhinoplasty is the distinction between subjective symptom improvement and objectively measurable airflow changes. Across the included studies, functional outcomes were predominantly assessed using validated patient-reported outcome measures (PROMs), such as the ROE, NOSE, and RHINO scores, all of which consistently demonstrated significant postoperative improvement. While these instruments are essential for capturing patient perception of nasal breathing and overall satisfaction, they primarily reflect symptom burden rather than quantifiable nasal airflow dynamics. Only a limited number of studies incorporated objective assessments, such as rhinomanometry, acoustic rhinometry, or internal nasal valve angle measurements, and even in these cases, methodologies and reporting standards varied considerably. Consequently, although preservation rhinoplasty appears to yield meaningful symptomatic relief, the current evidence base does not uniformly demonstrate corresponding objective superiority in airflow parameters. Symptom improvement and measurable airflow enhancement, while related, are not interchangeable endpoints; patient-reported breathing comfort may improve due to subtle structural optimization, reduced edema, or altered airflow patterns that are not fully captured by standard objective tests. Therefore, claims of functional superiority should be interpreted with caution. Future prospective studies integrating both standardized PROMs and objective airflow measurements at predefined postoperative intervals will be essential to more definitively determine whether preservation techniques confer measurable physiological advantages in addition to perceived symptomatic benefit.

### Future Perspectives and Challenges

Despite the significant advancements in preservation rhinoplasty, several challenges and future directions remain. One major limitation is the lack of standardized protocols for preservation techniques, leading to variability in outcomes across different surgical centers. Future research should focus on establishing evidence-based guidelines for technique selection, patient candidacy, and long-term follow-up [5].

Additionally, advancements in surgical instrumentation and imaging technology are expected to further refine preservation rhinoplasty techniques. The integration of minimally invasive approaches, including ultrasonic instrumentation and endoscopic-assisted procedures, may enhance precision while reducing tissue trauma [45]. Furthermore, the growing field of regenerative medicine presents exciting opportunities for cartilage preservation and tissue engineering, potentially allowing for biologically enhanced grafts that maintain long-term structural integrity without the need for extensive cartilage harvest. Importantly, the current evidence base remains limited in size and is largely observational, with incomplete reporting of key patient-centered outcomes (including PROM-based satisfaction in several studies). These factors reduce the conclusiveness of existing findings and underscore the need for larger, prospective, ideally comparative studies with standardized reporting to confirm and better generalize current observations. Specifically, well-designed comparative studies directly evaluating open structural rhinoplasty versus preservation and hybrid techniques within comparable patient populations will be essential to determine relative effectiveness, particularly in complex cases. Until such data are available, conclusions regarding superiority in selected patient groups should be interpreted cautiously and viewed as hypothesis-generating rather than definitive.

In conclusion, preservation rhinoplasty has emerged as a transformative approach in modern nasal surgery, offering distinct advantages in maintaining nasal framework integrity and optimizing aesthetic outcomes. The integration of hybrid techniques, improved patient selection strategies, and technological advancements will continue to shape the future of this field. Continued innovation, coupled with rigorous clinical validation, will be essential in establishing preservation rhinoplasty as a widely accepted standard in nasal surgery.

## Limitations

This study has several limitations that should be considered when interpreting the results. First, the inherent heterogeneity among the included studies—in terms of surgical techniques, patient demographics, follow-up durations, and outcome measures—limits direct comparability and introduces potential bias. The concept of "preservation rhinoplasty" itself lacks a universally accepted definition, with techniques and modifications often varying significantly between surgeons. As such, conducting a review on this evolving and ambiguous surgical category presents inherent challenges, and comparisons between studies must be interpreted with caution. The variation in outcome reporting, with some studies emphasizing aesthetic improvements while others focused on functional changes, further complicates synthesis. Another important limitation is the inconsistent reporting of anatomical complexity. Key parameters—such as the presence of severe septal deviations, deformities extending to the K-point, or cases requiring total septal reconstruction—were rarely defined in a standardized way. Because these factors strongly influence revision rates, surgical difficulty, and overall satisfaction, the lack of uniform severity descriptors limits the ability to compare preservation and structural techniques reliably across studies. Moreover, the majority of included studies relied on subjective patient-reported outcomes, such as ROE or NOSE scores, while only a few incorporated objective airflow or anatomical measurements. While PROMs provide important patient-centered perspectives on perceived functional and aesthetic benefit, they do not substitute for dedicated, standardized psychological outcome measures (e.g., validated assessments of body image, self-esteem, or broader quality-of-life constructs). The limited and inconsistent use of such psychological instruments in the available preservation rhinoplasty literature restricts conclusions regarding psychosocial impact beyond symptom and satisfaction reporting. This lack of standardized, validated outcome measures limits the ability to perform robust cross-study comparisons. In terms of evidence quality, the absence of randomized controlled trials (RCTs) and the reliance on retrospective cohort designs lower the overall strength of evidence. Although methodological quality was assessed using the NOS and LOE frameworks, the predominance of observational data restricts the ability to draw definitive conclusions about long-term efficacy and complication rates. Another notable limitation is the short duration of follow-up in several studies, which may obscure late-onset complications such as dorsal irregularities, airway compromise, or progressive structural weakening. That said, in the case of closed preservation rhinoplasty, both aesthetic and functional outcomes observed after the first postoperative month are generally considered stable and reliable, as significant edema and healing have typically subsided by that point [45]. Finally, selection bias is likely, as patients chosen for preservation techniques often have favorable anatomy (e.g., minor deviations, intact dorsum), which may lead to an overestimation of success rates and reduce the generalizability of findings to more complex or revision cases. The heterogeneity of outcome instruments and incomplete statistical reporting prevented any valid quantitative synthesis. Standardization of reporting with uniform outcome sets—such as ROE and NOSE with means and SD at prespecified postoperative intervals—will be essential to enable future meta-analyses. Additionally, most included studies were Level III cohort analyses, which restrict the strength of inference. The predominance of retrospective designs further limits causal interpretation and underscores the need for prospective, ideally randomized, studies with standardized outcome measures and long-term follow-up.

## Conclusion

Preservation rhinoplasty continues to evolve as a contemporary alternative to traditional structural techniques and may offer satisfactory aesthetic outcomes, maintenance of dorsal contour integrity, and favorable patient-reported satisfaction in appropriately selected patients. The findings of this systematic review suggest that preservation approaches might contribute to stable nasal framework support and relatively low reported revision rates; however, these observations are largely derived from lower evidence levels and should be interpreted with caution. Hybrid techniques may further expand the potential applicability of preservation concepts, particularly in cases where selective structural reinforcement is required in addition to dorsum conservation.

Careful patient selection remains central to optimizing outcomes. Based on the currently available literature, preservation rhinoplasty may represent a viable option in selected patients, rather than a universally superior approach. Its comparative advantages over traditional structural techniques cannot yet be definitively established. Additional high-quality, prospective studies with standardized objective and patient-reported outcome measures, as well as longer follow-up periods, will be necessary to clarify functional durability, refine indications, and better define its role within modern nasal surgery. As surgical techniques and technologies continue to advance, preservation rhinoplasty could play an increasingly relevant, though still evolving, role in contemporary practice.

## Supplementary Information

Below is the link to the electronic supplementary material.

Supplementary file1 (DOCX 17 KB)
