# Supplementary material for: Shaping the Future of Nasal Surgery: A Systematic Review of Preservation Rhinoplasty
Source: Aesthetic Plast Surg. 2026 May 19;50(13):4962–78. doi: 10.1007/s00266-026-05853-9 (PMC13433595; doi:10.1007/s00266-026-05853-9)
Supplement: Supplementary file 1 [file 266_2026_5853_MOESM1_ESM.docx]

**11. Supplementary Digital Content**

**Supplementary Digital Content 1.** Search strings in PubMed/MEDLINE, Web of Science and EMBASE databases with the respective number of search results.

| **Database** | **Search String** | **Results** |
| --- | --- | --- |
| PubMed/MEDLINE | ("Preservation rhinoplasty"[Title/Abstract] OR "push-down technique"[Title/Abstract] OR "let-down  technique"[Title/Abstract] OR "structural preservation rhinoplasty"[Title/Abstract]) AND  ("indications"[Title/Abstract] OR "patient selection"[Title/Abstract] OR "outcomes"[Title/Abstract] OR  "complications"[Title/Abstract] OR "adverse effects"[Title/Abstract] OR "future  developments"[Title/Abstract] OR "trends"[Title/Abstract] OR "innovations"[Title/Abstract] OR "combined  approach"[Title/Abstract] OR "hybrid technique"[Title/Abstract] OR "AI"[Title/Abstract] OR "artificial  intelligence"[Title/Abstract] OR "machine learning"[Title/Abstract] OR "perioperative patient  management"[Title/Abstract] OR "digital planning"[Title/Abstract] OR "computer-assisted  surgery"[Title/Abstract]) | 87 |
| Web of Science | TS=("Preservation rhinoplasty" OR "push-down technique" OR "let-down technique" OR "structural preservation rhinoplasty") AND TS=("indications" OR "patient selection" OR "outcomes" OR "complications" OR "adverse effects" OR "future developments" OR "trends" OR "innovations" OR "combined approach" OR "hybrid technique" OR "AI" OR "artificial intelligence" OR "machine learning" OR "perioperative patient management" OR "digital planning" OR "computer-assisted surgery") | 90 |
| EMBASE | ('preservation rhinoplasty'/exp OR 'preservation rhinoplasty' OR 'push-down technique' OR 'let-down technique' OR 'structural preservation rhinoplasty')  AND  ('indication'/exp OR 'indications' OR 'patient selection' OR 'outcome'/exp OR 'outcomes' OR 'complication'/exp OR 'complications' OR 'adverse effect'/exp OR 'adverse effects' OR 'future developments' OR 'trend'/exp OR 'trends' OR 'innovation'/exp OR 'innovations' OR 'combined approach' OR 'hybrid technique' OR 'artificial intelligence'/exp OR 'AI' OR 'machine learning'/exp OR 'machine learning' OR 'perioperative care'/exp OR 'perioperative patient management' OR 'digital planning' OR 'computer assisted surgery'/exp OR 'computer-assisted surgery') | 99 |

**Supplementary Digital Content 2.** Level of Evidence (LOE) quality assessment of studies included.

| **Author** | **DOI** | **Title** | **Sample size** | **Study design** | **LOE** |
| --- | --- | --- | --- | --- | --- |
| Öztürk et al. | 10.1007/s00266-020-01660-y | Push-Down Technique Without Osteotomy: A New Approach | 62 | Cohort study | 3 |
| Kosins et al. | 10.1093/asj/sjz107 | Decision Making in Preservation Rhinoplasty: A 100 Case Series With One-Year Follow-Up | 153 | Cohort study | 3 |
| Ferraz et al. | 10.1055/s-0041-1725154 | Indications for Preservation Rhinoplasty: Avoiding Complications | N/A | Technical note | N/A |
| Öztürk | 10.1093/asj/sjaa436 | Semi–Let-Down and Semi–Push-Down Preservation Techniques: Maintaining the Intactness of the Distal Region | 64 | Cohort study | 3 |
| Tuncel et al. | 10.1093/asj/sjac069 | Dorsal Preservation Surgery: A Novel Modification for Dorsal Shaping and Hump Reduction | 25 (22 primary, 3 secondary rhinoplasty cases) | Cohort study | 3 |
| Öztürk et al. | 10.1097/SCS.0000000000008553 | Hybrid Preservation Rhinoplasty: Combining Mix-Down and Semi Let-Push Down Techniques | 36 | Cohort study | 3 |
| Kosins | 10.1093/asj/sjac074 | Preservation Rhinoplasty: Open or Closed? | 162 | Cohort study | 3 |
| Stergiou et al. | 10.1016/j.bjps.2021.05.073 | A multivariate analysis after preservation rhinoplasty (PR) - a prospective study | 30 | Cohort study | 3 |
| Urs et al. | 10.37897/RMJ.2022.2.7 | Risks and complications in rhinoplasty. A comparative study in structural vs preservation rhinoplasty | 100 | Cohort study | 3 |
| Tapan et al. | 10.1097/SAP.0000000000003681 | Let-Down Rhinoplasty in Patients With Cleft Lip Nose | 10 | Cohort study | 3 |
| Anco et al. | 10.1097/GOX.0000000000004972 | Preservation Rhinoplasty: A New Approach to Mestizo Noses | 14 | Cohort study | 3 |
| Scafati et al. | 10.1097/PRS.0000000000011307 | Low Strip Unifying Hybrid Rhinoseptoplasty: A Novel Classification in Dorsal Preservation Surgery | 190 | Cohort study | 3 |
| Jin et al. | 10.1097/PRS.0000000000010472 | Dorsal Preservation Rhinoplasty in Asian Hump Noses | 9 | Case series | 4 |
| *DOI, Digital object identifier; N/A, Not applicable; LOE, Level of Evidence* | | | | | |

**Supplementary Digital Content 3.** Newcastle-Ottawa Scale (NOS) quality assessment of studies included.

| **DOI** | **Author** | **Title** | **Study design** | **Selection of study cohort** | **Comparability of cohorts** | **Assessment of outcomes** | **Final score** |
| --- | --- | --- | --- | --- | --- | --- | --- |
| 10.1007/s00266-020-01660-y | Öztürk et al. | Push-Down Technique Without Osteotomy: A New Approach | Cohort study | 3 | 1 | 2 | 6 |
| 10.1093/asj/sjz107 | Kosins et al. | Decision Making in Preservation Rhinoplasty: A 100 Case Series With One-Year Follow-Up | Cohort study | 3 | 1 | 2 | 6 |
| 10.1055/s-0041-1725154 | Ferraz et al. | Indications for Preservation Rhinoplasty: Avoiding Complications | Technical note | N/A | N/A | N/A | N/A |
| 10.1093/asj/sjaa436 | Öztürk | Semi–Let-Down and Semi–Push-Down Preservation Techniques: Maintaining the Intactness of the Distal Region | Cohort study | 3 | 1 | 2 | 6 |
| 10.1093/asj/sjac069 | Tuncel et al. | Dorsal Preservation Surgery: A Novel Modification for Dorsal Shaping and Hump Reduction | Cohort study | 3 | 1 | 2 | 6 |
| 10.1097/SCS.0000000000008553 | Öztürk et al. | Hybrid Preservation Rhinoplasty: Combining Mix-Down and Semi Let-Push Down Techniques | Cohort study | 2 | 1 | 2 | 5 |
| 10.1093/asj/sjac074 | Kosins | Preservation Rhinoplasty: Open or Closed? | Cohort study | 3 | 1 | 2 | 6 |
| 10.1016/j.bjps.2021.05.073 | Stergiou et al. | A multivariate analysis after preservation rhinoplasty (PR) - a prospective study | Prospective study | 3 | 1 | 2 | 6 |
| 10.37897/RMJ.2022.2.7 | Urs et al. | Risks and complications in rhinoplasty. A comparative study in structural vs preservation rhinoplasty | Cohort study | 3 | 1 | 2 | 6 |
| 10.1097/SAP.0000000000003681 | Tapan et al. | Let-Down Rhinoplasty in Patients With Cleft Lip Nose | Cohort study | 3 | 1 | 2 | 6 |
| 10.1097/GOX.0000000000004972 | Anco et al. | Preservation Rhinoplasty: A New Approach to Mestizo Noses | Cohort study | 3 | 1 | 2 | 6 |
| 10.1097/PRS.0000000000011307 | Scafati et al. | Low Strip Unifying Hybrid Rhinoseptoplasty: A Novel Classification in Dorsal Preservation Surgery | Cohort study | 3 | 1 | 2 | 6 |
| 10.1097/PRS.0000000000010472 | Jin et al. | Dorsal Preservation Rhinoplasty in Asian Hump Noses | Retrospective case series | 2 | 1 | 2 | 5 |
| *DOI, Digital object identifier; N/A, Not applicable; LOE, Level of Evidence* | | | | | | | |
